# Microbiological patterns of bacterial bloodstream infections and predictors of kidney dysfunction at a tertiary hospital in Zambia

**DOI:** 10.11604/pamj.2026.54.29.52096

**Published:** 2026-06-04

**Authors:** John Nzobokela, David Chisompola, Elijah Chinyante, Luswepo Namwinga, Lucky Kalyapu, Nanjela Chindima

**Affiliations:** 1 Department of Cardiovascular Science and Metabolic Diseases, Livingstone Center for Prevention and Translational Science, Livingstone, Zambia,; 2 Department of Pathology, Ndola Teaching Hospital, Ndola, Zambia,; 3 Department of Biomedical Sciences, Chikankata College of Biomedical Sciences, Mazabuka, Zambia,; 4 Department of Biomedical Sciences, Ndola College of Biomedical Sciences, Ndola, Zambia

**Keywords:** Kidney dysfunction, sepsis, Gram-negative bacteria, platelets, red cell distribution width

## Abstract

**Introduction:**

bloodstream infections (BSIs) are a significant cause of morbidity and mortality and are frequently complicated by kidney dysfunction. Evidence on the prevalence, predictors, and associated microbiological patterns in Zambia remains scarce. This study evaluated the burden of kidney dysfunction, its laboratory predictors, and blood culture isolate patterns at Ndola Teaching Hospital (NTH)

**Methods:**

a retrospective cohort study was conducted at NTH from January to December 2025. Data were extracted from the DISA laboratory database and patient registry records. Kidney dysfunction was defined as an estimated glomerular filtration rate (eGFR) of less than 60 mL/min/1.73 m^2^ at admission. Descriptive statistics summarized demographic and microbiological characteristics. Logistic regression analyses were performed to identify independent predictors of kidney dysfunction.

**Results:**

among 232 patients with confirmed BSIs, 58.6% (n = 136; 95% CI: 52.1-64.9) had kidney dysfunction at admission. The majority were aged 18-39 years (37.9%) and ≥60 years (34.9%), and 51.3% were female. Gram-negative organisms predominated (67.1%), particularly Enterobacterales (52.2%), with Enterobacter agglomerans (15.1%), Klebsiella pneumonia (10.8%), and Escherichia coli (8.2%) most common. Gram-positive organisms accounted for 32.3%, mainly Staphylococcus aureus (16.8%). Multidrug-resistant isolates comprised 58.2%. Independent predictors of kidney dysfunction at admission included age 40-59 years (AOR 2.39; 95% CI 1.01-5.61) and age ≥60 years (AOR 5.62; 95% CI 2.41-13.08) compared with patients aged 18-39 years. Compared with patients with pre-existing chronic kidney disease (CKD), those without CKD had markedly lower odds of kidney dysfunction at admission (AOR 0.007; 95% CI 0.00-0.11). Among laboratory markers, a lower hemoglobin-to-RDW ratio (AOR 0.04; 95% CI 0.03-0.85) and lower platelet count (AOR 0.995; 95% CI 0.994-0.998) were also independently associated with kidney dysfunction at admission.

**Conclusion:**

kidney dysfunction at admission is highly prevalent among adults with BSIs at NTH and is independently associated with older age, pre-existing CKD, lower hemoglobin-to-RDW ratio, and thrombocytopenia. The predominance of multidrug-resistant Gram-negative pathogens highlights the importance of early renal assessment, risk stratification, and strengthened antimicrobial stewardship in resource-limited settings.

## Introduction

Bloodstream infections (BSIs) remain a major cause of morbidity and mortality globally, with a disproportionately high burden in low- and middle-income countries [1,2]. A critical but often under-recognized complication of BSIs is kidney dysfunction, which is associated with prolonged hospitalization, increased need for intensive care, and higher mortality rates [3].

The clinical impact of BSIs is further worsened by the rise of multi-drug-resistant (MDR) organisms [4]. Evidence from upper-middle-income countries in Africa and the Western Pacific indicates that pathogens such as *Enterobacteriaceae, Acinetobacter baumannii*, and *Staphylococcus aureus* are commonly implicated in severe BSIs and are linked to adverse clinical outcomes [1,2].

In sub-Saharan Africa, the true burden of BSI-related kidney dysfunction is likely underestimated because of limited access to blood culture diagnostics and inconsistent renal monitoring [5]. However, routinely available laboratory markers may offer practical tools for early risk stratification in resource-limited settings.

In Zambia, data on the prevalence of kidney dysfunction among patients with BSI, its predictors, and the spectrum of causative organisms remain scarce. This study therefore aimed to determine the prevalence of kidney dysfunction in patients with bloodstream infections, identify its clinical and laboratory predictors, and describe the microbiological profile of bloodstream isolates at Ndola Teaching Hospital (NTH) in Zambia.

## Methods

**Study design and setting:** this was a laboratory-based, single-center retrospective cohort study conducted at NTH, a tertiary referral hospital in Ndola, Copperbelt Province, Zambia. NTH is a major referral center for specialized medical care and serves an average of 29,485 patient admissions per year, providing comprehensive clinical and diagnostic services, including microbiology and renal function testing.

The study reviewed data from patients with confirmed bloodstream infections diagnosed between January and December 2025. Data extraction was conducted after ethical approval, from 1^st^ November, 2025 to 10^th^ January, 2026, using records retrieved from the laboratory information system (LIS)-DISA database.

**Study participants and eligibility:** the study included adult patients (≥18 years) with at least one laboratory-confirmed bloodstream infection and documented renal function results (serum creatinine and/or estimated glomerular filtration rate) at the time of hospital admission. Patients were excluded if they had incomplete or missing laboratory records, duplicate blood culture isolates from the same infectious episode, or organisms classified as contaminants. Contaminants were defined according to Clinical and Laboratory Standards Institute (CLSI) guidelines, including common skin commensals (e.g., *coagulase-negative staphylococci, Corynebacterium spp*., *Bacillus spp*., and *Micrococcus spp*.) isolated from a single blood culture without clinical evidence of infection, or organisms inconsistent with the patient´s clinical presentation. Additionally, patients with sickle cell disease or malignancies that could affect complete blood count parameters were excluded to avoid confounding of hematological predictors of kidney dysfunction.

**Population:** all 582 positive blood culture entries on the DISA*Lab were reviewed. After excluding incomplete records, duplicate isolates, contaminants, and patients with sickle cell disease or malignancies, 232 participants with confirmed bloodstream infections were included.

**Study variables:** the primary outcome of this study was kidney dysfunction, defined as an estimated glomerular filtration rate (eGFR) of less than 60 mL/min/1.73 m^2^ at hospital admission. Independent variables included demographic factors age (categorized into three clinically meaningful groups: 18-39 years, 40-59 years, and ≥60 years, reflecting increasing risk of kidney dysfunction with advancing age and consistent with prior studies on infection-related renal impairment [6,7]) and sex, clinical factors (care unit at the time of bloodstream infection), laboratory parameters (hemoglobin, platelets (PLT), red cell distribution width (RDW), white blood cells (WBC)). Absolute neutrophil, lymphocyte, and monocyte counts, as well as calculated inflammatory ratios such as hemoglobin-to-red cell distribution width ratio (HB/RDW), derived neutrophil-to-lymphocyte ratio (dNLR), neutrophil-to-platelet ratio (NPR), and systemic immune-inflammation index (SII), common comorbidities included hypertension, diabetes mellitus, HIV infection and pre-existing chronic kidney dysfunction (CKD), defined as a documented history of CKD prior to the current hospital admission, based on prior medical records and clinician diagnosis and microbiological factors such as; type of bloodstream isolate, including Gram-negative versus Gram-positive bacteria, *Enterobacterales*, non-fermentative bacteria, and multidrug-resistant organisms. These variables were evaluated to determine predictors of kidney dysfunction and to describe the microbiological profile of bloodstream infections in the study population.

**Data analysis:** data were collected, cleaned, coded, and analyzed using Stata version 15. Descriptive statistics, including proportions, frequencies, medians, and interquartile ranges (IQR), were used to summarize study variables. The Shapiro-Wilk test was applied to assess the normality of continuous variables. Associations between kidney dysfunction and independent variables were evaluated using bivariate analysis, and variables with p<0.05 were included in a multivariate logistic regression model to identify independent predictors. Results were reported as odds ratios (OR) with 95% confidence intervals (CI), with statistical significance set at p<0.05.

**Ethical considerations:** ethical approval was obtained from the Mulungushi University School of Medicine and Health Sciences Research Ethics Committee (IRB: 00012281, FWA: 0002888, Ref. No.: SMHS-MU1-2025-37) on 17^th^ March, 2025, and the National Health Research Ethics Board (REF: NHRA-2246/20/05/2025) on 5^th^ June, 2025. Authorization to conduct the study was granted by Ndola Teaching Hospital. All data were de-identified to ensure confidentiality, and no information that could identify participants was collected. Written or verbal consent was waived by the ethics committees. The study was conducted in accordance with the Declaration of Helsinki and Good Clinical Practice guidelines.

## Results

**Descriptive and microbiological characteristics:** a total of 232 patients with laboratory-confirmed bloodstream infection were included in the study. The largest proportion of participants were aged 18-39 years (37.9%), followed by those aged ≥60 years (34.9%), and 27.2% were between 40-59 years. Females constituted 51.3% of the cohort, while males made up 48.7%. Kidney dysfunction at admission was present in 58.6% (136/232) of patients, whereas 41.4% (96/232) had no evidence of renal impairment. Hospital location at the time of bloodstream infection refers to the ward or unit where the patient was physically admitted when the blood culture was collected, reflecting the setting in which infection was first suspected and evaluated. Among the participants, 50.4% of patients were in the general wards, 37.5% in the emergency/admission unit, and 12.1% in the intensive care unit. Regarding comorbidities, hypertension was present in 11.2% (26/232) of patients, diabetes mellitus in 4.3% (10/232), pre-existing chronic kidney disease in 27.2% (63/232), and HIV infection in 15.9% (37/232). Gram-negative bacteria accounted for 67.1% of all bloodstream isolates, with *Enterobacterales* comprising 52.2% of total pathogens. The most frequent *Enterobacterales* were *Enterobacter agglomerans* (15.1%), *Klebsiella pneumonia* (10.8%), and *Escherichia coli* (8.2%). Non-fermentative Gram negative organisms represented 15.5% of isolates, predominantly *Acinetobacter baumannii* (8.6%) and *Pseudomonas spp*. (6.0%). Gram-positive bacteria constituted 32.3% of isolates, led by *Staphylococcus aureus* (16.8%), followed by *Streptococcus pneumonia* (6.5%) and *Enterococcus faecalis* (4.3%). Multidrug-resistant organisms (MDROs) were identified in 58.2% of isolates, while 41.8% were non-MDRO pathogens (Table 1).

**Table 1:** demographic characteristics, clinical features, and microbiological profile of bloodstream infection isolates among adult patients with laboratory-confirmed bloodstream infections at Ndola Teaching Hospital, Ndola, Zambia, January-December 2025 (N = 232)

Variable	Value (n=232)	%
**Age, years**		
18-39	88	37.9
40-59	63	27.2
≥60	81	34.9
**Sex**		
Males	113	48.7
Females	119	51.3
**Kidney dysfunction**		
No	96	41.4
Yes	136	58.6
**Care unit**		
Emergency/admission	87	37.5
General wards	117	50.4
Intensive care unit	28	12.1
**Bloodstream isolates**		
Gram-negative bacteria	157	67.1
*Enterobacterales*	121	52.2
*Citrobacter species*	4	1.7
*Enterobacter agglomerans*	35	15.1
*Enterobacter cloacae*	3	1.3
*Escherichia coli*	19	8.2
*Klebsiella oxytoca*	4	1.7
*Klebsiella pneumoniae*	25	10.8
*Morganella morganii*	6	2.6
*Proteus mirabilis*	4	1.7
Salmonella species	16	6.9
*Serratia marcescens*	2	0.9
*Yersinia enterocolytica*	2	0.9
*Hafnia alvei*	1	0.4
Non-fermentative	36	15.5
*Acinetobacter baumannii*	20	8.6
Burkholderia species	2	0.9
*Pseudomonas spp*.	14	6
Gram-positive bacteria	75	32.3
*Streptococcus pneumoniae*	15	6.5
*Viridans streptococcus*	6	2.6
*β-haemolytic streptococcus*	5	2.1
*Enterococcus faecalis*	10	4.3
*Staphylococcus aureus*	39	16.8
**Multidrug-resistant organisms**		
No	97	41.8
Yes	135	58.2

**Relation between kidney dysfunction and clinical and laboratory characteristics:** patients with kidney dysfunction were significantly older than those without renal impairment (p<0.001). The prevalence of kidney dysfunction was lowest among individuals aged 18-39 years (26.5%), slightly higher in those aged 40-59 years (27.9%), and highest in patients aged ≥60 years (45.6%). Among comorbidities, hypertension (17.6% vs. 2.1%, p<0.001) and pre-existing chronic kidney disease (45.6% vs. 1%, p<0.001) were significantly more frequent in patients with kidney dysfunction (Table 2).

**Table 2:** association between kidney dysfunction and clinical characteristics among adults with laboratory-confirmed bloodstream infections (N = 232)

Variable	Kidney dysfunction		P-value
	No	Yes	
**Age years, n (%)**			<0.001*
18-39	52 (54.2)	36 (26.5)	
40-59	25 (26)	27 (27.9)	
≥60	19 (19.8)	62 (45.6)	
**Sex, n (%)**			**0.741**
Females	48 (50)	71 (52.2)	
Males	48 (50)	65 (47.8)	
**Bloodstream isolates, n (%)**			**0.693**
Gram-negative bacteria	64 (66.7)	94 (69.1)	
Gram-positive bacteria	32 (33.3)	42 (30.9)	
**Bacteria group, n (%)**			**0.240**
*Enterobacterales*	45 (46.9)	76 (55.9)	
Non-fermenters	19 (19.8)	12 (12.5)	
Gram-positive cocci	32 (33.3)	43 (31.6)	
**Care unit, n (%)**			**0.721**
Emergence/admission	35 (36.5)	52 (38.2)	
General wards	51 (53.1)	66 (48.5)	
Intensive care unit	10 (10.4)	18 (13.2)	
**Multidrug-resistant organisms, n (%)**			**0.189**
No	45 (46.9)	52 (38.2)	
Yes	51 (53.1)	84 (61.8)	
**Hypertension, n (%)**			<0.001*
No	94 (97.9)	112 (84.4)	
Yes	2 (2.1)	24 (17.6)	
**HIV status, n (%)**			**0.910**
Negative	81 (84.4)	114 (83.2)	
Positive	15 (15.6)	23 (16.8)	
**Diabetes mellitus, n (%)**			**0.234**
No	94 (97.9)	129 (94.9)	
Yes	2 (2.1)	7 (5.1)	
**Pre-existing CKD, n (%)**			<0.001*
No	95 (99)	74 (54.4)	
Yes	1 (1)	62 (45.6)	

* Significant p-value <0.05; P-values were calculated using Chi-square test; CKD: chronic kidney disease

With respect to hematological parameters, median HB levels were significantly lower in patients with kidney dysfunction at admission (8.3 (6.3-11.1) g/dL) compared with those without renal impairment (10.1 (7.7-13.1) g/dL) (p < 0.001). RDW was higher in the kidney dysfunction group (16.4 (14.9-19.7)) than in patients without kidney dysfunction (14.9 (13.6-18.1)) (p = 0.002), resulting in a significantly lower HB/RDW ratio (0.50 (0.32-0.69) vs. 0.66 (0.45-0.94), p<0.001). Platelet counts were reduced in patients with kidney dysfunction (139 (81.2-266) x10^9^/L) compared with those without kidney dysfunction (189 (102-297) x10^9^/L) (p = 0.012). Additionally, patients with kidney dysfunction had higher absolute neutrophil counts (ANC) (7.24 (3.90-14.5) x10^9^/L vs. 5.84 (3.21-9.43) x10^9^/L, p = 0.009), derived neutrophil-to-lymphocyte ratio (dNLR) (4.44 (2.36-8.34) vs. 2.99 (1.88-5.10), p = 0.005), and neutrophil-to-platelet ratio (NPR) (0.05 (0.02-0.09) vs. 0.03 (0.01-0.06), p = 0.001) than those without kidney dysfunction (Table 3).

**Table 3:** association between kidney dysfunction and laboratory characteristics among adults with laboratory-confirmed bloodstream infections (N = 232)

Variable	Kidney dysfunction		P-value
	No	Yes	
WCC, median (IQR)	7.97 (5.23-12.24)	9.19 (5.07-16.97)	0.083
HB, median (IQR)	10.1 (7.7-13.1)	8.3 (6.3-11.1)	<0.001**
RDW, median (IQR)	14.9 (13.6-18.1)	16.4 (14.9-19.7)	0.002**
HB/RDW, median (IQR)	0.66 (0.45-0.94)	0.50 (0.32-0.69)	<0.001
PLT, median (IQR)	189 (102-297)	139 (81.2-226)	0.012**
ANC, median (IQR)	5.84 (3.21-9.43)	7.24 (3.90-14.5)	0.009**
ALC, median (IQR)	1.17 (0.68-1.84)	1.04 (0.63-1.45)	0.194
AMC, median (IQR)	0.41 (0.22-0.66)	0.41 (0.20-0.79)	0.709
DNLR, median (IQR)	2.99 (1.88-5.10)	4.44 (2.36-8.34)	0.005**
SII, median (IQR)	786 (349-2665)	947 (465-2251)	0.456
NPR, median (IQR)	0.03 (0.01-0.06)	0.05 (0.02-0.09)	0.001**

** significant p-value <0.05; P-values were calculated using Mann-Whitney U test; WCC: white blood cell count; HB: hemoglobin; RDW: red cell distribution width; PLT: platelet count; ANC: absolute neutrophil count; ALC: absolute lymphocyte count; AMC: absolute monocyte count; dNLR: derived neutrophil-to-lymphocyte ratio; SII: systemic immune-inflammation index; NPR: neutrophil-to-platelet ratio; IQR: interquartile range

**Predictors associated with kidney dysfunction in logistic regression:** in unadjusted logistic regression analysis, older age, lower HB, lower platelet counts, higher ANC, higher WCC, and lower HB/RDW were associated with increased odds of kidney dysfunction. Patients aged 40-59 years had more than twice the odds of kidney dysfunction compared with those aged 18-39 years (OR 2.19; 95% CI 1.13-4.24; p = 0.015), while those aged ≥60 years had nearly fivefold higher odds (OR 4.71; 95% CI 2.41-9.18; p < 0.009). Pre-existing CKD was also strongly associated with kidney dysfunction in univariable analysis (p<0.001).

After adjustment for potential confounders, older age remained an independent predictor of kidney dysfunction. Compared with patients aged 18-39 years, those aged 40-59 years had 2.39-fold higher odds (AOR 2.39; 95% CI 1.01-5.61; p = 0.045), and patients aged ≥60 years had more than fivefold higher odds (AOR 5.62; 95% CI 2.41-13.08; p<0.001). Pre-existing CKD remained strongly associated with kidney dysfunction (AOR 0.007; 95% CI 0.00-0.11; p<0.001).

Among laboratory markers, a lower HB/RDW ratio independently predicted kidney dysfunction (AOR 0.04; 95% CI 0.03-0.85; p = 0.039), while lower platelet counts were also significant (AOR 0.995; 95% CI 0.994-0.998; p = 0.02). Other variables, including sex, hypertension, WCC, HB, ANC, and NPR, were not independently associated with kidney dysfunction after adjustment (Table 4).

**Table 4:** predictors associated with kidney dysfunction in logistic regression

Variables	OR (95% Cl)	P value	AOR (95 % Cl)	P-value
**Age, years**				
18-39	Ref		Ref	
40-59	2.19 (1.13-4.24)	0.015*	2.39 (1.01-5.61)	0.045*
≥60	4.71 (2.41-9.18)	0.009*	5.62 (2.41-13.08)	<0.001*
**Sex**				
Females	Ref		Ref	
Males	1.10 (0.64-1.84)	0.741	0.861 (0.42-1.72)	0.674
**Hypertension**				
No	Ref	0.002*	Ref	
Yes	10.07 (2.32-43.72)		0.40 (0.25-6.43)	0.519
**Pre-existing CKD**		<0.001*		
No	0.013 (0.002-0.093)		0.007 (0.00-0.11)	<0.001*
Yes	Ref		Ref	
WCC	1.04 (1.00-1.08)	0.02*	0.97 (0.851-1.105)	0.697
HB	0.869 (0.79-0.94)	0.001*	1.22 (0.95-1.572)	0.103
RDW	1.03 (0.98-1.09)	0.199		
HB/RDW	0.14 (0.05-0.39)	<0.001*	0.04 (0.03-0.85)	0.039*
PLT	0.99 (0.99-1)	0.035*	0.995 (0.994-0.998)	0.02*
ANC	1.06 (1.01-1.10)	0.005*	1.09 (0.94-1.26)	0.211
DNLR	1.03 (0.99-1.08)	0.122		
NPR	586 (6.2-55048)	0.006*	3.32 (0.02-534.5)	0.643

* significant p-value <0.05, p-values were calculated using the logistic regression analysis; OR: odds ratio; AOR: adjusted odds ratio; CI: confidence interval; WCC: white blood cell count; HB: hemoglobin; RDW: red cell distribution width; PLT: platelet count; ANC: absolute neutrophil count; dNLR: derived neutrophil-to-lymphocyte ratio; NPR: neutrophil-to-platelet ratio; CKD: chronic kidney disease

**Criteria for determination of multidrug-resistant organism status:** multidrug-resistant organism (MDRO) status was determined based on phenotypic antimicrobial susceptibility testing results. An isolate was classified as MDRO if it showed non-susceptibility (resistant or intermediate) to at least one antimicrobial agent in three or more distinct antimicrobial classes. Resistance was assessed across the following major antimicrobial classes, depending on organism type (Table 5).

**Table 5:** operational definition and criteria used to determine multidrug-resistant organism (MDRO) status among bacterial bloodstream isolates

Variable	Coding	Criteria	Major antimicrobial classes and representative drugs
MDRO	0 = non-MDRO	Susceptible to ≥2 major antimicrobial classes	-
	1 = MDRO	Resistant to ≥1 agent in ≥3 major antimicrobial classes	For Gram-negative bacteria (Enterobacterales, Non-fermenters): 1. β-lactams (penicillins, cephalosporins) - ampicillin, amoxicillin-clavulanate, ceftriaxone, ceftazidime, cefepime; 2. carbapenems - meropenem, imipenem; 3. fluoroquinolones - ciprofloxacin, levofloxacin; 4. aminoglycosides - gentamicin, amikacin, tobramycin; 5. sulfonamides - trimethoprim-sulfamethoxazole; 6. tetracyclines - doxycycline, tigecycline. For Gram-positive bacteria (Staphylococcus, Enterococcus): 1. β-lactams (penicillins, cephalosporins) - oxacillin/methicillin, amoxicillin; 2. Glycopeptides - vancomycin, teicoplanin; 3. macrolides - erythromycin; 4. fluoroquinolones - ciprofloxacin, levofloxacin; 5. aminoglycosides - gentamicin (high-level resistance)

Determination of multidrug-resistant organism status. MDRO classification was based on resistance to at least one antimicrobial agent in three or more major antimicrobial classes according to standard antimicrobial resistance definitions. MDRO, multidrug-resistant organism; ≥: greater than or equal to

## Discussion

This study demonstrates a high prevalence of kidney dysfunction (58.6%) among adults with bloodstream infections at Ndola Teaching Hospital, highlighting the substantial renal burden associated with BSI in a tertiary care setting. Gram-negative bacteria predominated, particularly *Enterobacterales* such as *Enterobacter agglomerans, Klebsiella pneumonia*, and *Escherichia coli*, which are clinically significant due to their frequent association with severe infections, healthcare-associated transmission, and multidrug resistance [8,9]. Gram-positive organisms, led by *Staphylococcus aureus*, also contributed substantially, with multidrug-resistant organisms comprising over half of all isolates, reflecting the growing challenge of antimicrobial resistance.

The high prevalence of kidney dysfunction aligns with previous reports where infection and sepsis are major contributors to acute kidney injury, with incidence estimates ranging from approximately 14% to over 60% depending on patient populations and definitions [10]. This underscores the substantial burden of renal involvement in systemic infections and highlights the importance of early renal monitoring [11].

The microbiological profile in this study, where Gram-negative bacteria accounted for 67.1% of bloodstream isolates, with *Enterobacterales* predominating and non-fermentative organisms such as *Acinetobacter baumannii* and *Pseudomonas spp*. also frequently isolated, alongside Gram-positive pathogens led by *Staphylococcus aureus*, is consistent with findings from other settings. In a large surveillance study reported that Gram-negative organisms comprised approximately two-thirds of bloodstream infection isolates, with *Escherichia coli, Klebsiella pneumonia*, and non-fermenters among the most common pathogens, while *S. aureus* remained a leading Gram-positive cause of BSI [12]. Similarly, a retrospective cohort of multidrug-resistant bloodstream infections found that Gram-negative bacteria such as *E. coli, K. pneumoniae, Pseudomonas spp*., and *Acinetobacter spp*. were the predominant isolates, with *S. aureus* also frequently recovered [13]. In a study from The Gambia, *S. aureus* and *Klebsiella spp*. were the most common bloodstream pathogens, with other *Enterobacterales* and non-fermentative organisms also present [14]. A similar distribution was observed in an Indian multicenter study where Gram-negative bacilli, including *E. coli, K. pneumoniae, Pseudomonas aeruginosa*, and *Acinetobacter spp*. accounted for a majority of BSI isolates [15]. These corroborative studies from diverse geographic regions underscore the global predominance of Gram-negative bacteria in BSIs and the persistent role of key pathogens such as *Enterobacterales, A. baumannii, Pseudomonas spp*., and *S. aureus* in bloodstream infection epidemiology. A recent systematic review and meta-analysis including studies from Africa and other low- and middle-income settings consistently identified *Escherichia coli, Klebsiella spp*., and *Staphylococcus aureus* as the leading causes of bacteremia, with high levels of resistance to third-generation cephalosporins and other commonly used antibiotics, underscoring their importance as priority pathogens for antimicrobial resistance surveillance and empiric therapy planning [16,17].

In multivariable analysis, older age was a strong independent predictor of kidney dysfunction at admission. Participants aged 40-59 years and ≥60 years had significantly higher odds of renal impairment compared with those aged 18-39 years. This is consistent with evidence that the likelihood of developing acute kidney injury increases with age, as age-related structural and functional renal decline and a higher burden of comorbidities predispose older adults to renal impairment in critical illness and sepsis [18]. A recent cohort study has shown that advancing age is independently associated with higher acute kidney injury (AKI) risk in critically ill and septic populations, with a stepwise increase in AKI incidence and kidney dysfunction among older age groups [19]. In our study, AKI was considered within this broader clinical context but was not directly diagnosed. Pre-existing chronic kidney disease at admission was also associated with kidney dysfunction compared with patients without a history of chronic kidney disease in our study. Prior studies similarly identify baseline chronic kidney disease as a major risk factor for poor renal outcomes during systemic infections and critical illness, reflecting reduced physiological reserve and susceptibility to further injury in patients with sepsis [20-22].

Among hematological markers, a lower HB/RDW ratio was independently associated with kidney dysfunction. Elevated RDW and related red cell distribution metrics have been linked with kidney dysfunction incidence and severity in critically ill or septic patients in multiple studies, suggesting that anisocytosis and erythrocyte turnover reflect systemic inflammation and impaired microvascular function in renal injury [22]. Similarly, lower platelet counts independently predicted kidney dysfunction. Thrombocytopenia has been reported as a predictor of kidney dysfunction in sepsis and HIV-associated sepsis cohorts, with lower platelet counts marking more severe systemic inflammation, endothelial dysfunction, and greater renal vulnerability [23]. In a prospective cohort of sepsis patients in Uganda, thrombocytopenia was independently associated with the development of kidney dysfunction [24]. In critically ill kidney dysfunction patients, reductions in platelet count have been correlated with greater adverse renal events and worse outcomes, including increased risk of major adverse kidney events and mortality [25].

While the precise mechanisms underlying kidney dysfunction in bloodstream infection remain complex and multifactorial, our findings highlight that both host factors (older age, chronic kidney disease at index admission) and readily available laboratory markers (HB/RDW ratio, platelet count) can help identify patients at high risk of renal impairment. However, due to the retrospective design and reliance on single-time-point serum creatinine measurements at admission, we were unable to reliably distinguish between acute kidney injury and chronic kidney disease. Consequently, the outcome in this study is best interpreted as kidney dysfunction at admission, with acute and chronic renal impairment considered possible underlying contributors rather than confirmed diagnoses. Prospective studies incorporating longitudinal kidney function assessments and biomarkers of early renal injury are needed to better delineate these pathways in patients with bloodstream infections.

**Strengths and limitations:** this study has several limitations. Its retrospective, single-center design may limit generalizability and is subject to incomplete or missing clinical records, which could have affected the accuracy of some variables. In addition, although the sample size was adequate to detect major associations, it may have limited statistical power to identify more subtle predictors. Despite these limitations, the study has important strengths. To our knowledge, this is the first study in the region to comprehensively examine the relationship between bloodstream infections and kidney dysfunction, providing novel and locally relevant evidence. The use of the DISA*Lab laboratory information system enabled robust capture of high-quality microbiological and hematological data, strengthening the reliability of our findings. Importantly, this study provides real-world evidence from a resource-limited setting, which is directly applicable to routine clinical practice. Our findings have potential to inform service delivery, guide early risk stratification, and support policy decisions related to the management of patients with kidney dysfunction and infections caused by multidrug-resistant organisms in similar settings.

## Conclusion

Kidney dysfunction at admission was highly prevalent among adults with bloodstream infections at Ndola Teaching Hospital, affecting nearly six in ten patients. Independent predictors included older age, pre-existing chronic kidney disease, lower hemoglobin-to-RDW ratio, and thrombocytopenia, highlighting patients at greatest risk. Gram-negative bacteria, particularly *Enterobacterales*, and multidrug-resistant organisms predominated among bloodstream isolates. These findings emphasize the need for early renal assessment, close monitoring of high-risk patients, and strengthened infection control and antimicrobial stewardship to reduce the burden of renal complications in resource-limited settings.

***What is known about this topic***

- *Bloodstream infections (BSIs) are a major cause of morbidity and mortality globally, particularly in sub-Saharan Africa;*
- *Kidney dysfunction is a common complication of severe infections and is associated with poor clinical outcomes;*
- *Gram-negative and multidrug-resistant organisms increasingly complicate the management of BSIs in low-resource settings*.

***What this study adds***

- *Demonstrates a high prevalence (58.6%) of kidney dysfunction at admission among adults with laboratory-confirmed BSIs at a tertiary hospital in Zambia;*
- *Identifies older age and lower platelet count as independent predictors of kidney dysfunction at admission using routinely available laboratory parameters;*
- *Provides updated local data on bloodstream isolate patterns, showing predominance of multidrug-resistant Gram-negative organisms, particularly Enterobacterales*.
